# Supplementary material for: Content-rich biological network constructed by mining PubMed abstracts
Source: BMC Bioinformatics. 2004 Oct 8;5:147. doi: 10.1186/1471-2105-5-147 (PMC528731; doi:10.1186/1471-2105-5-147)
Supplement: Additional File 2 — The original results of the above study (non-essential files are deleted to keep the file size under the limit set by BMC bioinformatics). [file 1471-2105-5-147-S2.bz2 › chilibotAdditionalFile2/dip05/83ID8798570E297/html/EPHB1_EFNB1.html]

 


 **EPHB1** and **EFNB1** 
  
Found 51 abstracts in PubMed, retrieved 05.  
 

 What does Google say? 
 PDF only 
| .edu only 

---

**Interactive relationship** (e.g. stimulation, inhibition, etc)

**Neutral relationship**- Alternatively, EphB  [ **EPHB1** ]  receptor signaling may act as a ligand density sensor and titrate signaling pathways that promote branch extension toward an optimal ephrin B1  [ **EFNB1** ]  concentration found at the TZ.  Ref: 12702655 Development, 2003

**Non-interactive relationship** (e.g. studied together, co-existance, homology, etc.)

- We propose that Xdsh, which is expressed in various tissues, is involved in EphB  [ **EPHB1** ]  and ephrin B  [ **EFNB1** ]  signaling related to regulation of cell repulsion via modification of RhoA activity.  Ref: 12574121 EMBO J, 2003
- EphB  [ **EPHB1** ]  receptors are expressed in a low to high DV gradient by retinal ganglion cells RGCs, and ephrin B1  [ **EFNB1** ]  is expressed in a low to high LM gradient in the tectum.  Ref: 12702655 Development, 2003
- We report that the EphB  [ **EPHB1** ]  receptor ligand, ephrin B1  [ **EFNB1** ] , may act bifunctionally as both a branch repellent and attractant to control the unique mechanisms in mapping the dorsal ventral DV retinal axis along the lateral medial LM axis of the optic tectum.  Ref: 12702655 Development, 2003
- In contrast, stimulation of Jurkat cells with the EphB  [ **EPHB1** ]  receptor ligand ephrin B1  [ **EFNB1** ]  does not cause Cbl phosphorylation.  Ref: 12794130 J Immunol, 2003
- A major recent advance is the demonstration that EphB  [ **EPHB1** ]  receptor forward signaling and ephrin B  [ **EFNB1** ]  reverse signaling mediate axon attraction to control dorsal ventral retinal mapping along the lateral medial tectal axis.  Ref: 12593983 Curr Opin Neurobiol, 2003
